# 

**Published:** 1899-02

**Authors:** 


### NOTICES.

### Fortieth Annual Meeting of the Northern Ohio Dental Association.

The fortieth annual meeting of the Northern Ohio Dental Association, will be held at Cleveland (Colonial Hotel), May 16, 17, and 18, 1899, beginning at 10 o'clock a. m., sharp, Tuesday, May 16th. The program is as follows: 1, Reading of Minutes; 2, Reports of Officers and Committees; 3, Reading of Papers and Discussion; 4, Election of Officers.

President's Address, Dr. L. P. Bethel, Kent; The Dentist in his Office, Dr. N. B. Acheson, Youngstown, discussion opened by Drs. H. F. Harvey, Cleveland, Frank Creager, Fremont; Report of Committee on President's Address, general discussion; The World and its Teeth (illustrated with stereopticon), Dr. W. H. Whitslar, Cleveland, discussion opened by Drs. J. R. Callahan, Cincinnati, C. R. Butler, Cleveland; Poem, "Oh, Give Us Back Our Tails," Dr. J. F. Siddall, Oberlin; A Symposium, Six short papers on the most interesting case in practice during the last two years—Denture with Almost an Entire Upper Lip Made and Attached, Dr. F. S. Whitslar, Youngstown—An Unerupted Cuspid, Dr. C. T. King, New London—Subject to be announced, Dr. J. F. Dougherty, Canton—Subject to be announced, Dr. J. G. Templeton, Pittsburg—A Case in Oral Surgery, Dr. D. E. Kelley, Ashtabula—Subject to be announced, Dr. F. L. Warren, Painesville—general discussion; Preparation of Proximal Cavities in Bicuspids and Molars (demonstrated by models), Dr. J. K. Douglas, Sandusky, discussion opened by Drs. W. H. Merritt, Norwalk, D. A. Allen, Toledo; Revision of Constitution and By-Laws; Some considerations Pertaining to the Filling of Teeth, Dr. F. W. Knowlton, Akron, discussion opened by Drs. Neil H. Bishop, Andover, Wm. T. Binzley, Napoleon; The Use of X-Rays in Dentistry, Dr. W. A. Price, Cleveland (Dr. Price will demonstrate his paper by using an X-Ray apparatus), discussion opened by Drs. W. H. Hersh, Piqua, L. E. Custer, Dayton; Voluntary Papers, general discussion.

Clinics will be given in the Dental College of Western Reserve University, as follows: 1, Symposium on Napkining, Drs. C. R. Butler, Cleveland; A. Terry, Norwalk; J. W. Lyder, Akron; E. J. Waye, Sandusky; H. Barnes, Cleveland; J. E. Robinson, Cleveland. 2, Symposium on the Extraction of the Lower Teeth, Drs. W. H. Fowler, Painesville; W. G. Ebersole, Cleveland; W. B. Connor, Akron; F. H. Waldron, Canal Dover; L. W. Ballard, Alliance; G. H. Wilson, Cleveland. 3, Cohesive Tinfoil Filling, Dr. H. L. Ambler, Cleveland. 4, Tin Demonstration, Dr. Corydon Palmer, Warren. 5, The Use of Incandescent Lamps to Control Dental Electrical Apparatus, Dr. W. H. Hersh, Piqua. 6, Clinic, L. E. Custer, Dayton. 7, Use of Sulphuric Acid in Canal Preparation, Dr. J. R. Callahan, Cincinnati. 8, Clinic, Dr. Hugh B. Mitchell, Canton. 9, Making Case-Richmond Crowns, Practical Cases, Dr. J. F. Stephan, Cleveland. 10, Gold Plating, Dr. W. H. Hayden, Youngstown. 11, The Process of Tipping Teeth by the Use of Solid Cusps, Dr. Everett M. Cook, Toledo. 12, Upper Plate (continuous gum), will use the Custer electric oven, Dr. W. T. Jackman, Cleveland. 13, Voluntary Clinics.

### EDITORIAL.

### THE DENTAL REGISTER,

A MONTHLY MAGAZINE DEVOTED TO THE INTERESTS OF THE

**DENTAL PROFESSION.**

*Terms Reduced to* $2.00 *Per Year. Single Copies,* 25 *Cents.*

**EDITED BY PROF. J. TAFT, D.D.S.**

—AND—

**Published by SAM'L A. CROCKER & CO., Ohio Dental and Surgical Depot.**

The volume commences in January and closes in December.

The REGISTER being issued monthly is always fresh, therefore Indispensable to the practitioner who desires to keep posted up with the new inventions and improvements which are daily developed. Neither expense nor effort will be spared to give value and variety to its contents. Articles will be illustrated with well executed engravings whenever they will add to the interest or assist to explanation.

Its extensive circulation and frequency of issue make it one of the BEST DENTAL ADVERTISING MEDIUMS in the United States.

All subscriptions must begin with January or July.

Local or special notices, five lines or less, will be inserted for $3 each insertion; over five lines, 50 cts. per line.

All advertising bills payable in advance, or at furthest, after the issue of the first number containing the advertisement. All transient advertisements to be paid for in advance.

☞CONTRIBUTIONS SOLICITED.

Exclusively Editorial Communications should be addressed to the Editor.

The latest number of the REGISTER may be ordered with or without other goods at any time, and all letters should be addre[illegible]

